# Round the Hospitals

**Published:** 1919-11-29

**Authors:** 


### ROUND THE HOSPITALS.

Nursing sisters on both sides of the Atlantic are gratified by the inclusion of two of their number in the investiture which marked the departure of the Prince of Wales from New York. The Prince, who was in naval uniform, decorated several distinguished officers in the American Army and Navy, and proceeded to bestow the Royal Red Cross on two nursing sisters who had displayed their quality in nursing the wounded. A classified list of honours bestowed since August 1914 and October 31 last has lately been published, from which it appears that the total number, exclusive of those conferred on members of the Royal Air Force, amounts to 249,387. The summary shows that the decoration of the Royal Red Cross, First Class, has been obtained by 869 persons, 450 of whom gained it for "services in the field," and 419 for "services in connection with the war." The number of those

**ROUND THE HOSPITALS**—(*continued*).

who have been made Associates of the Royal Red Cross during this period is not given.

The Queen of Spain is studying the methods pursued at Infant Welfare Centres with a view to introducing work of this kind in Spain, where infant mortality is extraordinarily high, and no preventive treatment of any kind is in operation. In the course of her investigations the Queen visited the North Islington Infant Welfare and Maternity Centre, in which wards have been initiated by the American Women's Club. Her Majesty went through every department of the Centre, and watched the nurses engaged in their ordinary routine with keen interest, asking many questions and making herself fully conversant with the reasons for the various things she saw in progress. A crowd of mothers and children were waiting for their turn to see the doctors, and the Queen displayed kindly sympathy with them, and herself attended four of the consultations. Before leaving she expressed the wish to Mrs. Keene, the Hon. Secretary, to see an analysis of the work accomplished, and to keep in touch with the Centre. It is an interesting example of the way in which women of all countries are beginning to draw together that an Infant Welfare Centre, founded in London, and largely supported from America, should be the model on which similar undertakings are to rise in Spain.

It is a real satisfaction to learn that the German chaplain who officiated in the gaol from which Miss Edith Cavell went out to her doom was, to use Mr. Gahan's words, "a sincere Christian man," and that he played his part towards her, not merely with decency, but with reverence. The account of her last hours contributed to a German paper (*Der Hochweg*) by Army Chaplain Paul de Seur is given on another page in translation. It contains details not hitherto published. It testifies to the deep impression made by Miss Cavell's character and demeanour on one of her "enemies." And it reinforces whatever has already been recounted of the dignity and fearlessness with which she met her end. The German comment on the proceedings is simple and to the point: "When I got home I felt sick at heart."

The Ladies' Association of the West London Hospital, Hammersmith, covers a wide field of work. It undertakes not only the supply of linen and other necessaries for the hospital, but also the Samaritan work and such other beneficent doings as the presentation of a new operating-table for the theatre at the cost of £100 and the provision of prizes for the nurses in training. Last year the linen alone cost £700. A large and influential meeting gathered on November 4 in the post-graduate college building at the hospital to receive the annual reports, and Miss Nevill, the matron, made a graceful speech, in which she exposed the needs of the hospital, and thanked the members of the League for their unfailing support, indispensable both to her and to the patients. Since last April the Samaritan Branch has assisted 320 patients. There is a capital library and visiting section, and the Needlework Guild supplies an immense variety of articles for patients who need clothing, and in some cases complete outfits. The live, spontaneous, and well-thought-out activities of the Association make it an excellent model for imitation.

The London County Council has made important improvements in the pay and organisation of their school nurses, who lately petitioned for a revision of their salaries. The staff as newly organised will consist of the superintendent of nurses, her six assistants, fifty-five nursing sisters, and 106 school nurses. The salaries are to be:—School nurses, from £157 to £181, including laundry allowance of £10, with uniform (valued at £10); school nursing sisters, from £181 to £205, including laundry allowance of £10, with uniform (valued at £10); assistant superintendents, from £207 to £244 10s., with uniform (valued at £10). In the great majority of cases the school nurses will be placed at the point next above their present salaries, with the result that each individual nurse will get a rise of about £10 a year, plus £10 a year laundry allowance. Temporary school nurses are also to receive higher pay, and will have uniform provided after six months' instead of as now after twelve months' service. The alterations will cost the Council from £4,000 to £5,000.

Armistice anniversary week was very suitably devoted in Newcastle and the district to collecting funds for the North-Eastern Local Centre of the College of Nursing. The project was warmly taken up locally, and, though it is too early to learn the total amount contributed, it is already certain the funds will benefit to a considerable extent. All the funds raised for the nurses under the Nation's Fund for Nurses will be kept distinct for the North-Eastern Local Centre. The patron of the Newcastle fund is the Lord Mayor of Newcastle, and the Lady Mayoress is President of the Collecting Committee, which includes Miss Brown (matron of the Royal Infirmary) and Miss Sandifer (matron of the military orthopædic hospital). Much of the work done by nurses is done in so quiet and unostentatious a manner that the public do not always recognise their debt for the devoted services performed by them both in war and in peace. Rich people are, unfortunately, chiefly brought into contact with nurses in the person of the really well paid private nurses who work under eminent specialists, and ignore the fact that these form a very inconsiderable minority in the profession.

Sir Arthur Stanley announced at the Liverpool Shipbrokers' Benevolent Society's dinner that £4,500 allocated to Red Cross work from the estate of the late Mr. J. H. Welsford would be devoted to form a fund for war nurses. The sum is to be invested in the names of Major Utting, Colonel Concannon, and Captain Mounsey. The interest

**ROUND THE HOSPITALS**—(*continued*).

is to be devoted annually by the trustees at their discretion in grants to distressed nurses, preference being given to nurses belonging to the district who were engaged on war work at home and abroad. So far as practicable, the operations of the Liverpool Fund are to be linked up with the Nation's Fund for Nurses, local management and local application of the funds being secured. The enormous nursing population in Liverpool, exceeding, perhaps, that of any other town except London, makes the gift particularly valuable. The Welsford Nurses' Fund will be a tower of strength to many nurses, now and in the future.

The movement to form a national memorial to Dr. Elsie Inglis has enlisted a great many notable people in its service, and a very interesting meeting has lately taken place, at which Lord Balfour of Burleigh, the Honorary Treasurer of the fund, urged the duty which devolved on British subjects to carry on the work in Serbia, to which this brave woman doctor had dedicated her life. A committee was formed to collect subscriptions for this purpose. The London office of the proposed national memorial is 120 Victoria Street, S.W. 1.

At Bolton the Guardians have utilised their War Hospital, which cost nearly £100,000, for the purposes of a general hospital, and admit private patients at a weekly charge of £2 2s. Patients are at liberty to have operations performed by their own practitioners, in which case the practitioner's fees are added, or by the surgeons at the institution without extra charge. In our judgment it is highly important to safeguard the position of nurses in municipal hospitals of this type. Well paid, properly housed, and with reasonable working hours, the staff is undoubtedly better off than if nursing the same class of patients in their own homes. We should welcome particulars of the conditions under which the Bolton nurses work in the new institution.

Many Canadian women are anxious to come to London next year in order to visit the graves of their relatives in this country and on the Continent. The Canadian Red Cross Society is already making preparations for them, and has secured a house in Prince's Gardens, where they will be received. When fitted up the house will accommodate fifty persons, and it is proposed to charge an inclusive fee of 7s. a day per head. Arrangements will also be made by the Red Cross for journeys to the graves abroad.

The York Guardians, after prolonged and at times arid discussion, have rejected the claims of their nurses to a fifty-six-hour week, and have declined to appoint the three additional nurses required for this purpose. The case was put in a nut-shell by one of the Guardians opposed to reform, who told the meeting there were two nurses to every thirty-eight patients, "and *yet* they were told there were not enough nurses." The Guardian who carried off the honours of the meeting, however, was the lady who insisted again and again that if nurses worked fewer hours they ought to get less pay. We confess we should like to see this economical individual wrestling for a few months during sixty hours out of every week with nineteen patients. The York ratepayers ought to put pressure on their representatives to provide that the infirmary is adequately staffed.

The authorities of Christ Church Hospital, New Zealand, appear to have treated their late lady superintendent, Miss Thurston, very badly. When she took up her military duties the board granted her leave of absence for the duration of the war. Her administrative gifts soon placed her at the head of the New Zealand Military Nursing Service, while her place at the hospital was filled by Miss Muir. Last August a movement (with which Miss Muir had nothing to do) started in the hospital to replace Miss Thurston by the temporary lady superintendent, and a letter was written to Miss Thurston, by the secretary, of a very ambiguous character, the gist of it being that the board was dissatisfied at the war lasting so long, and was anxious either that Miss Thurston should at once return to the hospital or that she should resign her office as matron. She replied, as it was her plain duty to do, that she could not leave her duties as Matron-in-Chief of the N.Z.M.N.S. The board has now by a narrow vote, nine against nine, with the chairman's casting vote deciding, cancelled the agreement to hold her post open, and appointed Miss Muir as matron. Remarks in the worst possible taste were made on Miss Thurston's war duties, and her "gay and glorious position" as matron-in-chief. The Christ Church board not merely gave her leave, but undertook to defray the difference between her war pay and her salary as matron. It is highly discreditable that a cabal formed against her in her absence should have been suffered to triumph. We note that more than one member of the board is heartily ashamed of the treatment accorded her.

The League of Red Cross Societies at Geneva announces that a ship filled with medical relief stores has been despatched for use in combating typhus in Poland, and ought by now to have arrived in Danzig. The supplies have been furnished by the Australian and American Red Cross. In answer to enquiries relating to the need for nurses to assist in combating the typhus epidemic in Poland, we have communicated with the B.R.C.S., and learn that, though it is understood that some nurses will be required by the League of Red Cross Societies in the near future, no particulars of requirements have yet been received from the League. It is, of course, out of the question for individual nurses to go out unless sent with an organised medical unit. The internationalising of the Red Cross Societies renders it possible to meet the needs of the countries affected with the least possible waste of energy, and private adventure parties can find no place in the organisation of relief.

**ROUND THE HOSPITALS—(*continued*).**

THE opening of the Nurses' Hostel at Stoke marks another step forward by releasing other accommodation for lying-in mothers at Plymouth. The institute already in existence consists of a maternity home, a hostel for orphans and motherless children, and a hostel for general and maternity district nurses. All three have become very crowded of late and the new hostel has been provided to relieve the congestion. The Mayor and Mayoress of Plymouth were present at the opening ceremony, when Dr. Down presided and explained that the hostel is an ideal place for the nurses and will accommodate from sixteen to twenty. He traced the rapid growth of the Alexandra Nursing Association, which, since 1907, has increased its staff from two to thirty nurses. St. Michael's Maternity Home is a first-rate training-school for midwives and has always done well in examinations. Already £10,000 has been contributed by the Navy League, through the representatives of Lady Beatty, towards the building of a much larger maternity home for Plymouth, the cost of which is estimated to exceed £50,000

---

THE end of a long period of mutual co-operation between English-speaking nurses from all parts of the Empire is marked by kindly letters of farewell to their friends from Miss Macdonald, Matron-in-Chief of the Canadian Army Nursing Service, and Miss Thurston, Matron-in-Chief of the New Zealand Military Nursing Service. Both ladies made their mark in this country as admirable administrators, courteous colleagues, and, in the highest sense of the words, good nurses.

---

FOUNDERS' DAY, the Festival of St. Luke, was observed with more than ordinary honour this year at the Royal Hampshire County Hospital, Winchester, as the first since the war. The matron and Committee invited all "old nurses" to a reunion, with the further delightful suggestion that they should spend the week-end at the hospital. This generous hospitality was rendered possible by the use of huts put up for military patients in the grounds. A very large number of nurses gathered from various parts of England, many of whom had not visited their Alma Mater since their war experiences, and nothing could have been more cordial than their reception. A special service was held in the chapel, and tea was served in the Beatrice Ward. A joyous party of over seventy sat down to supper. Some were, of course, prevented by their duties from remaining the night, but the huts received a large number of guests, whose holiday was enhanced by ideal weather. Among the improvements which excited much interest is the new electrical department, with apparatus recently installed by Messrs. Newton and Wright. The whirlpool baths, high-frequency apparatus, and a Bergonic chair were presented by Lady Cooper of Hursley Park. The Basingstoke Cottage Hospital has been presented with the old apparatus, still in very good condition.

---

NOTTINGHAM County and City chose as their war memorial a scheme for the reconstruction and extension of the General Hospital, and the sum collected so far has reached the handsome proportions of £84,640, including £15,000 allocated for this purpose from the British Red Cross Society. One of the first extensions to be carried out is a new nurses' home. The Castle site close to the hospital has been selected as most suitable for the home, and as it looks out over the Castle grounds, the nurses will have a fair prospect. There were buildings on this site which accommodated phthisical patients, but these have now been demolished and the patients have been moved to other quarters by the local health authorities. Nottingham nurses are not only to enjoy good accommodation suited to their requirements in the near future, they already receive better pay. The extra cost entailed by raising salaries stands at £230 in the report. The ordinary expenditure of the hospital has enormously increased, and the income has not kept pace with it, but the Board rightly insists that the profession should be adequately remunerated for their arduous work, and that if the hospital is to retain an efficient nursing staff these extra salaries must be paid.

